# Effects of Tannin‐Rich Forages ( *Lespedeza bicolor* and *Polygonum viviparum* ) Mixed With *Medicago sativa* on In Vitro Rumen Fermentation

**DOI:** 10.1002/fsn3.71015

**Published:** 2025-10-14

**Authors:** Jieqi Zhang, Yongsheng He, Zifan Chen, Fujiang Hou, Ding Guo, Jing Wang

**Affiliations:** ^1^ State Key Laboratory of Herbage Improvement and Grassland Agro‐Ecosystems, College of Pastoral Agriculture Science and Technology Lanzhou University Lanzhou Gansu P.R. China

**Keywords:** alfalfa, ammonia, condensed tannins, forage utilization, methane, mixing ratio

## Abstract

Ruminant production is currently facing many challenges, and thus it is essential to develop native sustainable feed resources. Alfalfa ( *Medicago sativa* ) is a high‐quality legume forage, but its rapid degradation in the rumen leads to bloating and more urine and methane production, resulting in nitrogen waste and environmental pollution. Mixing alfalfa with tannin‐rich forages is an effective way to improve rumen fermentation. Therefore, to utilize tannin‐rich forages such as *Lespedeza bicolor* and *Polygonum viviparum* as a partial replacement for alfalfa in the feed, three in vitro fermentation experiments were performed. In Experiments 1, 2, and 3, *L. bicolor* leaves, *P. viviparum* leaves, and *P. viviparum* bulbils were mixed with alfalfa at different ratios (0:100, 20:80, 40:60, 60:40, 80:20, and 100:0), respectively. Gas and methane production, fermentation parameters (pH, ammonia, microbial protein, and in vitro dry matter digestibility), and volatile fatty acid concentrations were determined to elucidate the effects of forage mixing on rumen fermentation. *L. bicolor* showed a more prominent effect on reducing gas and methane production than *P. viviparum* when mixed with alfalfa. When the mixing ratio of tannin‐rich forages and alfalfa was below the threshold of 40:60, the overall fermentation of the mixed substrates was improved compared to that of alfalfa alone. These findings will support the application of *L. bicolor* and *P. viviparum* in ruminant diets to improve practical livestock production and provide a specific forage use strategy in arid and semiarid grasslands and alpine meadows.

## Graphical abstract

Mixing alfalfa with tannin‐rich forages is an effective way to improve rumen fermentation. *Lespedeza bicolor* showed a more prominent effect on reducing gas and methane production than *Polygonum viviparum* when mixed with alfalfa. When the mixing ratio of tannin‐rich forages and alfalfa was below the threshold of 40:60, the overall fermentation of the mixed substrates was improved compared to that of alfalfa alone.

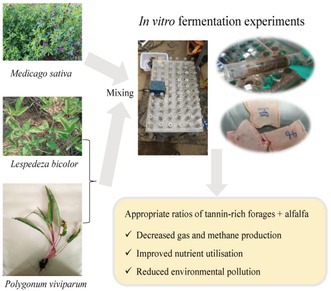

## 1 Introduction

Currently, ruminant production faces many challenges such as high greenhouse gas emissions, low utilization rate of forage nitrogen, and environmental pollution. Thus, developing new sustainable feed resources is urgent (Halmemies‐Beauchet‐Filleau et al. 2018). Alfalfa ( *Medicago sativa* ) is a high‐quality legume widely used in ruminant production due to its high yield, good palatability, and nutrient richness (Feng et al. 2022). However, when alfalfa is consumed in large quantities by ruminants, proteins in alfalfa are rapidly degraded by rumen microorganisms, resulting in bloating (Lagrange et al. 2021; Li et al. 2021). Moreover, a portion of the nitrogen and energy produced by rumen fermentation is released into the environment in the form of urine and methane (CH_4_), resulting in nitrogen waste and environmental pollution (Ghelichkhan et al. 2018).

The degradation of alfalfa protein in the rumen could be reduced by mixing tannin‐rich forages (TRF) in the feed (Kumar 2011). Tannins, especially condensed tannins (CT), are a class of secondary metabolites widely distributed in many plants (Min et al. 2003). Many studies have shown that TRF have positive effects on rumen fermentation. Tannins in forages can bind to proteins, reduce protein degradation, effectively inhibit bloating in ruminants, reduce ammonia (NH_3_‐N) production, increase protein flows to small intestines, and improve nitrogen utilization (MacAdam and Villalba 2015; Huang et al. 2018). For example, alfalfa mixed with CT‐containing *Broussonetia papyrifera* at appropriate ratios exhibited higher degradable dry matter (DM) content and released less gas compared with alfalfa alone (Li et al. 2021). A second strategy to reduce alfalfa protein degradation in the rumen is introducing CT‐producing genes into alfalfa leaves through genetic transformation (Kumar 2011). Studies have reported the introduction of CT into alfalfa by combined expression of a MYB family transcription factor and anthocyanidin reductase for the conversion of anthocyanidin into CT monomers (Xie et al. 2006). However, it will take a long time to produce commercial CT‐containing cultivars. Therefore, mixing alfalfa with TRF is a more effective and feasible way in livestock production.

*Lespedeza bicolor* and *Polygonum viviparum* are CT‐rich forages. *L. bicolor* is a protein‐rich legume forage adapted to arid and semiarid regions due to its excellent drought resistance (Zhang et al. 2025). *P. viviparum* is an important forage distributed widely in alpine meadows, and its leaves and bulbils are rich in proteins. In particular, its bulbils are palatable to livestock (Cui et al. 2016). However, neither of the TRF has been fully exploited in current livestock production.

To explore the feasibility of using TRF as a partial replacement for alfalfa in feeding, three in vitro fermentation experiments were performed. *L. bicolor* leaves, *P. viviparum* leaves, and *P. viviparum* bulbils were mixed with alfalfa at different ratios in Experiments 1, 2, and 3, respectively. Gas and CH_4_ production, fermentation parameters, and volatile fatty acid (VFA) concentrations were determined. Specifically, we hypothesized that (1) alfalfa mixed with tannin‐rich forages may produce better fermentation effects than alfalfa alone; (2) different tannin‐rich forage materials may have various effects on in vitro fermentation since the effects may depend on tannin content and sources. The results of this study will support the application of TRF ( *L. bicolor* and *P. viviparum* ) in ruminant diets and provide a specific forage use strategy in arid and semiarid grasslands and alpine meadows.

## 2 Materials and Methods

### 2.1 Experimental Materials

Forage materials were collected from the following locations: (1) Fresh leaves of *L. bicolor* were collected in a grassland near the Huanxian Grassland Agricultural Research Station of Lanzhou University (37.13° N, 106.82° E, altitude 1650 m) in June 2017. (2) Fresh leaves and bulbils of *P. viviparum* were collected from an alpine meadow in the Jinqiang River region in the northeast of the Qinghai‐Tibetan Plateau (37.23° N, 102.62° E, altitude 3147 m) in July 2021. (3) Alfalfa was collected from a farm in Lanzhou (35.57° N, 104.11° E, altitude 1966 m) in June 2017 and July 2021. After the forages were collected, they were placed in ice boxes and immediately brought back to the laboratory, freeze‐dried, ground up, passed through a 1‐mm mesh sieve and stored in the dark for later use.

Rumen fluid was collected as described below: For Experiment 1, rumen fluid was obtained from four ruminally fistulated 4‐year‐old healthy yellow cattle with similar growth conditions (body weight = 570 ± 20.8 kg) prior to the morning feeding. For Experiments 2 and 3, rumen fluid was collected from three 1.5‐year‐old small‐tailed Han sheep with similar growth conditions (body weight = 49.8 ± 1.97 kg). Prior to the morning feeding, a hard plastic pipe was carefully placed into the sheep's mouth, and a stomach tube connecting to a vacuum sampler was inserted into the rumen. The ingredients and chemical composition of the diets are listed in Table S1. These animals had free access to water throughout the experiments. After the fluid sampling, rumen fluid was combined, filtered through four layers of cheesecloth, transferred to a pre‐warmed thermos flask filled with carbon dioxide, and brought back to the laboratory. The rumen fluid was then kept in a water bath at 39°C for later use. All procedures on the yellow cattle and small‐tailed Han sheep followed the guidelines and protocols approved by the Animal Ethics Committee of Lanzhou University.

### 2.2 Chemical Composition

The forage materials were analyzed following the methods of the AOAC (1995) to determine organic matter by the muffle furnace incineration (ID 967.05) and crude protein (CP) by the Kjeldahl method (ID 984.13). Neutral detergent fiber (NDF) and acid detergent fiber (ADF) contents were analyzed as described by Van Soest et al. (1991). The CT content in forages was determined using the acid butanol assay (Hagerman 2011).

### 2.3 In Vitro Fermentation

Three in vitro fermentation experiments were conducted. *L. bicolor* leaves, *P. viviparum* leaves, and *P. viviparum* bulbils were mixed with alfalfa at different ratios (0:100, 20:80, 40:60, 60:40, 80:20, and 100:0) in Experiments 1, 2, and 3, respectively. Each treatment was assigned a name, and the details are shown in Table 1. The letters “A”, “B”, and “C” represent *L. bicolor* leaves, *P. viviparum* leaves, and *P. viviparum* bulbils, respectively. The numbers (0, 20, 40, 60, 80, and 100) following each letter (A, B, and C) indicate the percentage of TRF in the substrates. For example, “A20” means 20% *L. bicolor* leaves and 80% alfalfa in the substrates.

**TABLE 1:** Experimental treatments in Experiments 1, 2, and 3.

	Fermentation substrates	Treatment names and mixing ratios					
		A0	A20	A40	A60	A80	A100
Experiment 1	*L. bicolor* leaves	0	20%	40%	60%	80%	100%
	Alfalfa	100%	80%	60%	40%	20%	0

		B0	B20	B40	B60	B80	B100
Experiment 2	*P. viviparum* leaves	0	20%	40%	60%	80%	100%
	Alfalfa	100%	80%	60%	40%	20%	0

		C0	C20	C40	C60	C80	C100
Experiment 3	*P. viviparum* bulbils	0	20%	40%	60%	80%	100%
	Alfalfa	100%	80%	60%	40%	20%	0

In Experiment 1, each treatment was set up with six replicates, and 400 mg of substrates ( *L. bicolor* leaves mixed with alfalfa) was weighed for each replicate. In Experiments 2 and 3, each treatment was set up with three replicates, and 300 mg of substrates ( *P. viviparum* mixed with alfalfa) was weighed for each replicate. These substrates were then sealed in nylon bags (300 mesh). Three blanks without substrates were used to correct gas production and other fermentation parameters in each experiment.

In vitro fermentation experiments were performed according to Makkar et al. (1995). Artificial rumen fluid was prepared as described by Menke and Steingass (1988). The artificial rumen fluid was mixed with the collected rumen fluid at a ratio of 2:1 (v:v) to create fermentation liquids, with carbon dioxide continuously supplied throughout the mixing process to maintain an anaerobic environment. Glass syringes (100 mL; Häberle, Baden‐Württemberg, Germany) were used as fermentation vessels, which were calibrated prior to use. Latex tubes were fitted to the syringes and sealed with clamps to ensure airtightness. The day before each experiment, the nylon bags containing the substrates were placed inside the syringes. The syringes were placed in a water bath at 39°C overnight with a shaking frequency of 100 cycles min^−1^.

For each replicate, 40 mL of the fermentation liquids was drawn for Experiment 1, and 30 mL each for Experiments 2 and 3. The liquids were injected into the fermentation vessels through the latex tubes. The vessels were then placed in a water bath at 39°C to start the fermentation. In Experiment 1, three replicates of each treatment were removed at 24 h, and the remaining replicates continued to ferment until 72 h. In Experiments 2 and 3, all replicates were taken out at 48 h.

### 2.4 Observed Variables

The observed variables, including gas and CH_4_ production, fermentation parameters, and VFA concentrations, were determined as described by the literature (Zhang et al. 2025). Briefly, gas production was recorded by measuring plunger displacement (mL) at time *t* during the fermentation (Experiment 1: *t* was 3, 6, 9, 12, 18, 24, 36, 48, 60, and 72 h; Experiments 2 and 3: *t* was 3, 7.5, 12, 24, and 48 h). Methane produced during the fermentation was analyzed using gas chromatography (GC, HP4890D; Agilent Co., Santa Clara, CA, USA). The collected nylon bags were washed with water until the effluent was clear, and then dried at 105°C to a constant weight. The in vitro dry matter digestibility (IVDMD) was calculated as the difference between the DM of plant materials before and after fermentation. The pH of the fermentation liquids was measured using a calibrated pH meter (PHSJ‐3F; Shanghai Precision Scientific Instrument Co., China). The microbial protein (MCP) content was determined using the Coomassie Brilliant Blue assay kit (Nanjing Jiancheng Bioengineering Institute, China). The NH_3_‐N content was determined using the phenol hypochlorite method (Weatherburn 1967). The concentrations of acetate, propionate, isobutyrate, butyrate, isovalerate, and valerate were measured using a GC instrument (ThermoQuest 8000top; Italia SpA, Rodano, Milan, Italy). The concentration of total VFA (TVFA) was calculated as the sum of the six VFA concentrations mentioned above. A detailed description of the method for determining methane and VFA concentrations is shown in Method S1.

### 2.5 Statistical Analysis

Statistical analysis was conducted using SPSS 20.0 software (IBM, NY, USA). A normality test was performed prior to analysis. Significant differences among treatments were analyzed using one‐way ANOVA with a significance level set at *p* < 0.05. We analyzed significant differences in gas and CH_4_ production, in vitro fermentation parameters, and VFA concentrations among treatments. The LSD post hoc test was used if the data satisfied normality, whereas the Kruskal–Wallis *H* (*K*) test was used if the data did not satisfy normality. Additionally, we analyzed linear correlations between the percentage of TRF in the substrates and observed variables based on linear regression.

## 3 Results

### 3.1 Chemical Composition

The chemical composition of the substrates is summarized in Table 2. In all three experiments, increasing the proportion of the TRF led to a progressive decrease in CP content in the substrates. As the TRF percentage increased, the CP content in the substrates decreased sharply in Experiments 1 and 3, whereas it decreased slightly in Experiment 2. Compared to *P. viviparum* , *L. bicolor* contained more NDF. Condensed tannin content varied significantly among these forage materials. *L. bicolor* leaves contained the highest level (250.2 g kg^−1^ DM), followed by *P. viviparum* leaves (181.4 g kg^−1^ DM), while *P. viviparum* bulbils had the lowest level (63.8 g kg^−1^ DM).

**TABLE 2:** Chemical composition (g kg^−1^ DM) of substrates in Experiments 1, 2, and 3.

	Treatment names					
Experiment 1	A0	A20	A40	A60	A80	A100
OM	880.8	888.8	896.8	904.8	912.9	920.9
CP	210.1	195.4	180.8	166.0	151.3	136.6
NDF	385.7	376.4	367.1	357.9	348.6	339.4
ADF	246.7	232.1	217.5	202.8	188.2	173.6
CT	0	50.0	100.1	150.1	200.2	250.2

Experiment 2	B0	B20	B40	B60	B80	B100
OM	890.3	900.4	910.5	920.5	930.6	940.7
CP	232.1	228.1	224.1	220.0	216.0	212.0
NDF	175.7	162.8	149.8	136.9	123.9	111.0
ADF	144.3	131.9	119.5	107.1	94.7	82.3
CT	0	36.3	72.6	108.8	145.1	181.4

Experiment 3	C0	C20	C40	C60	C80	C100
OM	890.3	903.9	917.6	931.2	944.9	958.5
CP	232.1	212.9	193.8	174.6	155.5	136.3
NDF	175.7	185.2	194.7	204.3	213.8	223.3
ADF	144.3	152.7	161.1	169.5	177.9	186.3
CT	0	12.8	25.5	38.3	51.0	63.8

*Note:* A0–A100, B0–B100, and C0–C100: The letters “A”, “B”, and “C” represent *L. bicolor* leaves, *P. viviparum* leaves, and *P. viviparum* bulbils, respectively. The numbers (0, 20, 40, 60, 80, and 100) following each letter (A, B, and C) indicate the percentage of tannin‐rich forages in the substrates.

Abbreviations: ADF, acid detergent fiber; CP, crude protein; CT, condensed tannins; NDF, neutral detergent fiber; OM, organic matter.

### 3.2 Gas and Methane Production

**FIGURE 1:**
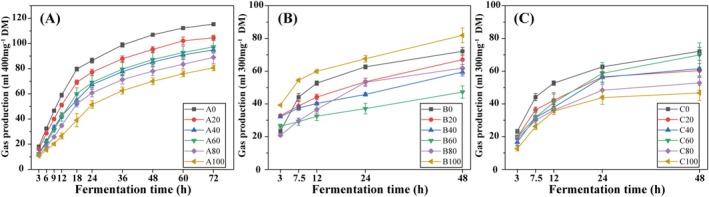
In vitro gas production of (A) *L. bicolor* leaves, (B) *P. viviparum* leaves, and (C) *P. viviparum* bulbils mixed with alfalfa. The data are mean ± SE of three replicates. A0–A100, B0–B100, and C0–C100: The letters “A”, “B”, and “C” represent *L. bicolor* leaves, *P. viviparum* leaves, and *P. viviparum* bulbils, respectively. The numbers (0, 20, 40, 60, 80, and 100) following each letter (A, B, and C) indicate the percentage of tannin‐rich forages in the substrates.

**FIGURE 2:**
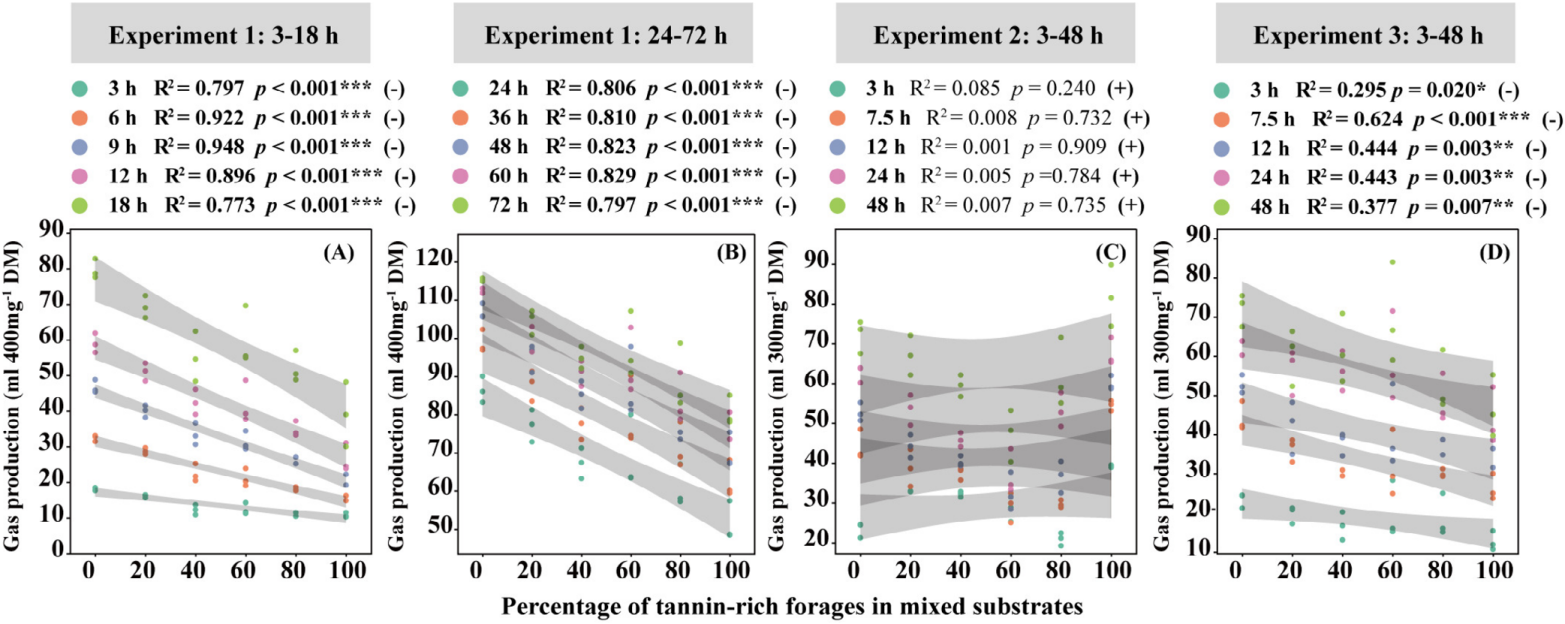
Linear correlations between the percentage of tannin‐rich forages in substrates and in vitro gas production (A) at 3–18 h in Experiment 1, (B) at 24–72 h in Experiment 1, (C) at 3–48 h in Experiment 2, and (D) at 3–48 h in Experiment 3. Linear regression was used as the test method. The shaded part of the figure is the band of the 95% confidence interval. (+) indicates a linear positive correlation; (−) indicates a linear negative correlation. *p < 0.05, **p < 0.01, ***p < 0.001.

Gas production of each treatment increased rapidly in the early fermentation stage and became stable in the later stage (Figure 1). In Experiments 1 and 3, there was a negative correlation between the gas production at each time point and the percentage of TRF in the mixed substrates (*p* < 0.05, Figure 2A,B,D). However, no significant linear relationship between the two factors was observed in Experiment 2 (Figure 2C, *p* > 0.05).

**FIGURE 3:**
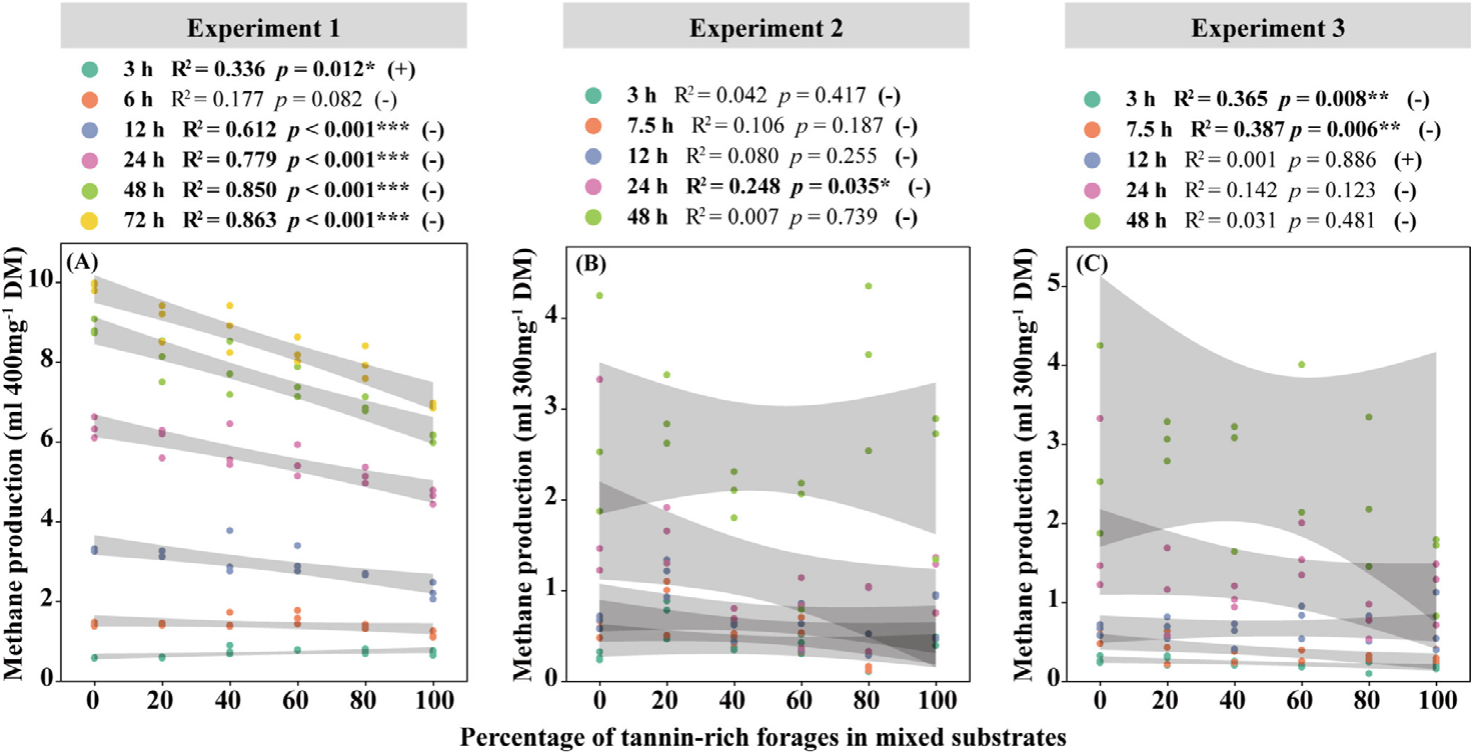
Linear correlations between the percentage of tannin‐rich forages in substrates and methane production at each time point (A) in Experiment 1, (B) in Experiment 2, and (C) in Experiment 3. Linear regression was used as the testing method. The shaded part of the figure is the band of the 95% confidence interval. (+) indicates a linear positive correlation; (−) indicates a linear negative correlation. *p < 0.05, **p < 0.01, ***p < 0.001.

As shown in Figure 3, there was a negative correlation between CH_4_ production and the proportion of *L. bicolor* leaves in the substrates after 12 h in Experiment 1 (*p* < 0.001). There was a negative correlation between CH_4_ production and the proportion of *P. viviparum* in the substrates at 24 h in Experiment 2 and at 3 and 7.5 h in Experiment 3 (*p* < 0.05). However, no significant linear relationship between the two factors was observed at other time points in Experiments 2 and 3 (*p* > 0.05).

In summary, only *L. bicolor* reduced both total gas production (Table 3) and CH_4_ yield (Table 4). In contrast, supplementation with *P. viviparum* leaves and its bulbils at varying levels did not significantly affect gas or CH_4_ production (Tables 3 and 4).

**TABLE 3:** Effects of tannin‐rich forages mixed with alfalfa on gas production during the in vitro fermentation.

		Treatments						*p*
Experiment 1		A0	A20	A40	A60	A80	A100	
Fermentation time	3 h	18.2 ± 0.27 a	16.1 ± 0.24 ab	12.3 ± 0.81 ab	12.4 ± 0.94 ab	11.1 ± 0.33 ab	10.7 ± 0.37 b	0.017*
	6 h	32.5 ± 0.50 a	28.7 ± 0.52 ab	22.5 ± 1.44 ab	21.1 ± 1.44 ab	18.1 ± 0.31 ab	15.3 ± 0.47 b	0.006**
	9 h	46.7 ± 1.07 a	40.0 ± 0.99 ab	33.4 ± 1.74 ab	31.3 ± 1.55 ab	25.8 ± 0.60 ab	20.2 ± 1.00 b	0.006**
	12 h	59.0 ± 1.57 a	51.1 ± 1.45 b	42.5 ± 2.02 c	41.9 ± 3.43 c	34.8 ± 1.25 d	26.5 ± 2.31e	< 0.001***
	18 h	79.7 ± 1.59 a	69.2 ± 1.79 ab	55.1 ± 4.05 ab	60.0 ± 4.80 ab	52.1 ± 2.51 ab	39.1 ± 5.25 b	0.012*
	24 h	86.5 ± 1.97 a	77.2 ± 2.43 ab	67.3 ± 2.31 ab	69.1 ± 5.50 ab	60.8 ± 3.14 ab	51.5 ± 2.97 b	0.013*
	36 h	98.8 ± 1.74 a	87.8 ± 2.29 b	77.6 ± 2.37 c	79.6 ± 5.37 bc	71.3 ± 3.45 cd	62.6 ± 2.77 d	< 0.001***
	48 h	106.9 ± 1.17 a	95.3 ± 2.15 ab	85.3 ± 2.08 ab	87.3 ± 5.29 ab	78.0 ± 3.54 ab	70.0 ± 2.70 b	0.014*
	60 h	112.3 ± 0.37 a	102.1 ± 3.09 ab	90.9 ± 1.92 ab	92.8 ± 5.05 ab	83.5 ± 3.78 ab	75.9 ± 2.33 b	0.012*
	72 h	115.4 ± 0.24 a	104.6 ± 1.88 b	94.9 ± 1.65 bc	97.4 ± 4.95 bc	89.0 ± 4.90 cd	80.6 ± 2.24 d	< 0.001***

Experiment 2		B0	B20	B40	B60	B80	B100	
Fermentation time	3 h	23.4 ± 1.08 ab	32.8 ± 0.03 ab	32.2 ± 0.38 ab	26.4 ± 1.18 ab	20.9 ± 0.93 b	39.3 ± 0.19 a	0.006**
	7.5 h	44.2 ± 2.17 b	38.7 ± 2.71 c	37.5 ± 0.89 c	29.1 ± 2.17 d	29.7 ± 0.54 d	54.5 ± 0.74 a	< 0.001***
	12 h	52.7 ± 1.32 b	44.2 ± 1.67 c	40.3 ± 0.76 cd	32.5 ± 2.73 e	36.7 ± 2.29 de	59.9 ± 1.04 a	< 0.001***
	24 h	62.6 ± 1.22 ab	53.5 ± 2.21 ab	45.8 ± 1.01 ab	37.1 ± 3.25 b	53.2 ± 2.47 ab	67.6 ± 2.02 a	0.007**
	48 h	72.2 ± 2.39 ab	67.1 ± 2.89 bc	59.5 ± 1.56 c	47.2 ± 3.76 d	61.9 ± 4.98 bc	81.9 ± 4.45 a	< 0.001***

Experiment 3		C0	C20	C40	C60	C80	C100	
Fermentation time	3 h	23.4 ± 1.08 a	19.7 ± 1.27 ab	16.7 ± 2.05 ab	19.8 ± 4.24 ab	18.7 ± 3.16 ab	12.7 ± 1.41 b	0.137
	7.5 h	44.2 ± 2.17 a	36.3 ± 1.70 b	31.6 ± 1.51 bc	31.8 ± 4.90 bc	30.0 ± 0.59 bc	26.2 ± 1.92 c	0.005**
	12 h	52.7 ± 1.32	42.2 ± 3.89	37.9 ± 1.70	40.9 ± 6.08	36.1 ± 1.30	35.6 ± 2.15	0.171
	24 h	62.6 ± 1.22	56.5 ± 3.36	56.2 ± 2.92	58.7 ± 6.62	48.4 ± 3.62	43.9 ± 4.18	0.100
	48 h	72.2 ± 2.39 a	60.4 ± 4.18 ab	61.6 ± 5.04 ab	69.9 ± 7.40 a	52.8 ± 4.41 b	46.7 ± 4.54 b	0.023*

*Note:* The unit of gas production in Experiment 1 is mL 400 mg^−1^ DM, whereas that in Experiments 2 and 3 is mL 300 mg^−1^ DM. The data are mean ± SE of three replicates. Different lowercase letters indicate significant differences (*p* < 0.05) among the treatments. A0–A100, B0–B100, and C0–C100: The letters “A”, “B”, and “C” represent *L. bicolor* leaves, *P. viviparum* leaves, and *P. viviparum* bulbils, respectively. The numbers (0, 20, 40, 60, 80, and 100) following each letter (A, B, and C) indicate the percentage of tannin‐rich forages in the substrates.

*p < 0.05, **p < 0.01, ***p < 0.001.

**TABLE 4:** Effects of tannin‐rich forages mixed with alfalfa on methane production during the in vitro fermentation.

		Treatments						*p*
Experiment 1		A0	A20	A40	A60	A80	A100	
Fermentation time	3 h	0.58 ± 0.004 b	0.59 ± 0.009 b	0.77 ± 0.065 a	0.78 ± 0.004 a	0.75 ± 0.034 a	0.71 ± 0.034 a	0.003**
	6 h	1.43 ± 0.028 ab	1.42 ± 0.016 ab	1.50 ± 0.113 ab	1.59 ± 0.098 a	1.36 ± 0.031 bc	1.16 ± 0.050 c	0.013*
	12 h	3.29 ± 0.021 a	3.17 ± 0.048 ab	3.13 ± 0.322 ab	3.01 ± 0.195 ab	2.68 ± 0.011 bc	2.24 ± 0.124 c	0.006**
	24 h	6.35 ± 0.153 a	6.02 ± 0.217 ab	5.81 ± 0.322 ab	5.49 ± 0.231 bc	5.15 ± 0.117 cd	4.62 ± 0.102 d	0.001**
	48 h	8.86 ± 0.110 a	8.05 ± 0.301 b	7.80 ± 0.389 b	7.46 ± 0.220 bc	6.92 ± 0.107 c	6.10 ± 0.062 d	< 0.001***
	72 h	9.90 ± 0.058 a	9.04 ± 0.276 b	8.85 ± 0.342 bc	8.27 ± 0.183 cd	7.97 ± 0.238 d	6.90 ± 0.036 e	< 0.001***

Experiment 2		B0	B20	B40	B60	B80	B100	
Fermentation time	3 h	0.27 ± 0.026 bc	0.71 ± 0.127 a	0.38 ± 0.025 b	0.41 ± 0.061 b	0.13 ± 0.015 c	0.45 ± 0.028 b	0.001**
	7.5 h	0.58 ± 0.055	0.87 ± 0.185	0.48 ± 0.031	0.52 ± 0.110	0.15 ± 0.014	0.66 ± 0.092	0.073
	12 h	0.66 ± 0.041 bc	1.16 ± 0.121 a	0.57 ± 0.065 bc	0.61 ± 0.150 bc	0.37 ± 0.078 c	0.79 ± 0.157 b	0.006**
	24 h	2.00 ± 0.664	1.62 ± 0.178	0.73 ± 0.038	0.78 ± 0.225	0.80 ± 0.236	1.14 ± 0.191	0.040*
	48 h	2.88 ± 0.709 ab	2.94 ± 0.225 ab	2.07 ± 0.148 ab	1.68 ± 0.445 b	3.50 ± 0.526 a	2.32 ± 0.492 ab	0.144

Experiment 3		C0	C20	C40	C60	C80	C100	
Fermentation time	3 h	0.27 ± 0.026	0.27 ± 0.036	0.23 ± 0.015	0.19 ± 0.006	0.20 ± 0.052	0.19 ± 0.020	0.182
	7.5 h	0.58 ± 0.055	0.43 ± 0.123	0.33 ± 0.047	0.30 ± 0.047	0.29 ± 0.016	0.32 ± 0.046	0.229
	12 h	0.66 ± 0.041	0.68 ± 0.079	0.59 ± 0.095	0.77 ± 0.123	0.62 ± 0.104	0.69 ± 0.223	0.926
	24 h	2.00 ± 0.664 a	1.14 ± 0.320 ab	1.06 ± 0.078 ab	1.63 ± 0.196 ab	0.76 ± 0.127 b	1.16 ± 0.232 ab	0.184
	48 h	2.88 ± 0.709 ab	3.04 ± 0.145 ab	2.65 ± 0.505 ab	5.26 ± 2.253 a	2.32 ± 0.551 ab	1.44 ± 0.311 b	0.246

*Note:* The unit of methane production in Experiment 1 is mL 400 mg^−1^ DM, whereas that in Experiments 2 and 3 is mL 300 mg^−1^ DM. The data are mean ± SE of three replicates. Different lowercase letters indicate significant differences (*p* < 0.05) among the treatments. A0–A100, B0–B100, and C0–C100: The letters “A”, “B”, and “C” represent *L. bicolor* leaves, *P. viviparum* leaves, and *P. viviparum* bulbils, respectively. The numbers (0, 20, 40, 60, 80, and 100) following each letter (A, B, and C) indicate the percentage of tannin‐rich forages in the substrates.

*p < 0.05, **p < 0.01, ***p < 0.001.

### 3.3 Fermentation Parameters and Volatile Fatty Acids

**FIGURE 4:**
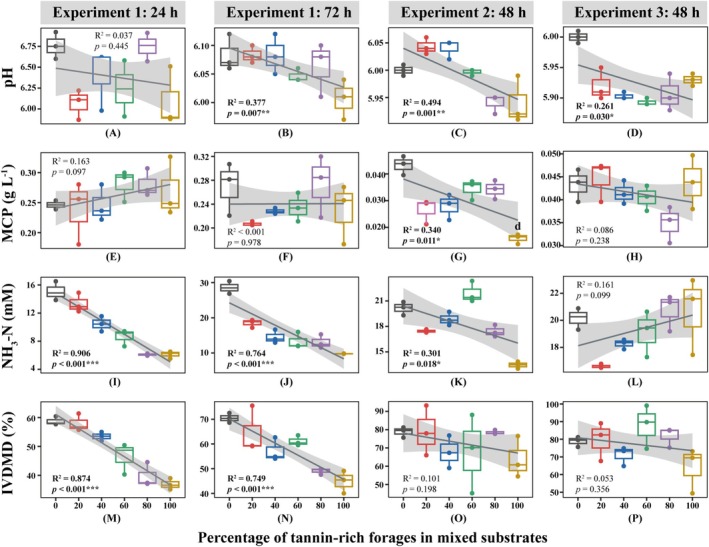
Linear correlations between the percentage of tannin‐rich forages in substrates and in vitro fermentation parameters. The shaded part of the figure is the band of the 95% confidence interval. The *p* and *R* ^2^ values in each panel were derived from linear regression. *p < 0.05, **p < 0.01, ***p < 0.001. IVDMD, in vitro dry matter digestibility; MCP, microbial protein; NH_3_‐N, ammonia.

As presented in Figure 4 and Table 5, the pH of the fermentation liquids was in the range of 5.50–7.00, and there was a negative correlation between the pH value and the proportion of TRF in the substrates (*p* < 0.05). In Experiment 1, the proportion of *L. bicolor* leaves in the substrates was negatively correlated with NH_3_‐N content or IVDMD (*p* < 0.001). However, in Experiments 2 and 3, no linear relationship was observed between IVDMD and CT content in the substrates (*p* > 0.05). In Experiment 2, the proportion of *P. viviparum* leaves in the substrates was negatively correlated with MCP or NH_3_‐N content (*p* < 0.05), whereas no such linear relationships were observed in Experiment 3 (*p* > 0.05).

**TABLE 5:** Effects of tannin‐rich forages mixed with alfalfa on in vitro fermentation parameters.

		Treatments						*p*
Experiment 1		A0	A20	A40	A60	A80	A100	
24 h	pH	6.76 ± 0.092	6.07 ± 0.103	6.41 ± 0.213	6.24 ± 0.193	6.75 ± 0.098	6.10 ± 0.207	0.050
	MCP (g L^−1^)	0.25 ± 0.004	0.24 ± 0.030	0.25 ± 0.018	0.28 ± 0.015	0.28 ± 0.014	0.27 ± 0.029	0.532
	NH_3_‐N (mM)	15.1 ± 0.78 a	13.4 ± 0.80 a	10.5 ± 0.62 b	8.6 ± 0.68 b	6.1 ± 0.08 c	6.1 ± 0.30 c	< 0.001***
	IVDMD (%)	58.7 ± 0.85 a	58.0 ± 1.74 a	53.5 ± 0.89 a	46.5 ± 3.12 b	39.7 ± 2.44 c	36.9 ± 1.13 c	< 0.001***
72 h	pH	6.08 ± 0.019	6.08 ± 0.009	6.08 ± 0.020	6.05 ± 0.007	6.06 ± 0.027	6.01 ± 0.020	0.108
	MCP (g L^−1^)	0.27 ± 0.026	0.21 ± 0.002	0.23 ± 0.003	0.23 ± 0.014	0.27 ± 0.030	0.23 ± 0.029	0.212
	NH_3_‐N (mM)	28.6 ± 1.04 a	18.5 ± 0.66 ab	14.5 ± 1.10 ab	13.3 ± 1.32 ab	13.1 ± 1.13 ab	9.8 ± 0.02 b	0.010*
	IVDMD (%)	70.5 ± 1.13 a	64.6 ± 5.43 ab	57.2 ± 2.75 ab	61.1 ± 1.21 ab	49.0 ± 0.73 ab	44.8 ± 2.65 b	0.016*

Experiment 2		B0	B20	B40	B60	B80	B100	
48 h	pH	6.00 ± 0.006	6.04 ± 0.009	6.04 ± 0.010	6.00 ± 0.003	5.94 ± 0.010	5.94 ± 0.025	0.011*
	MCP (g L^−1^)	0.04 ± 0.002 a	0.03 ± 0.003 c	0.03 ± 0.003 bc	0.03 ± 0.002 b	0.03 ± 0.002 b	0.02 ± 0.001 d	< 0.001***
	NH_3_‐N (mM)	20.2 ± 0.46 b	17.4 ± 0.11 c	18.9 ± 0.46 b	21.9 ± 0.73 a	17.4 ± 0.40 c	13.4 ± 0.24 d	< 0.001***
	IVDMD (%)	78.8 ± 1.64	79.1 ± 7.86	67.7 ± 5.13	67.8 ± 12.41	78.5 ± 0.68	63.9 ± 6.55	0.466

Experiment 3		C0	C20	C40	C60	C80	C100	
48 h	pH	6.00 ± 0.006 a	5.92 ± 0.015 ab	5.90 ± 0.003 ab	5.89 ± 0.003 b	5.91 ± 0.018 ab	5.93 ± 0.006 ab	0.036*
	MCP (g L^−1^)	0.04 ± 0.002 a	0.04 ± 0.003 a	0.04 ± 0.001 ab	0.04 ± 0.002 ab	0.03 ± 0.002 b	0.04 ± 0.003 a	0.101
	NH_3_‐N (mM)	20.2 ± 0.46 a	16.6 ± 0.09 b	18.3 ± 0.21 ab	19.1 ± 0.98 ab	20.7 ± 0.79 a	20.7 ± 1.66 a	0.038*
	IVDMD (%)	78.8 ± 1.64	79.7 ± 6.31	71.0 ± 3.13	87.8 ± 7.11	81.8 ± 3.28	64.0 ± 7.40	0.094

*Note:* Different lowercase letters indicate significant differences (*p* < 0.05) among growth stages. A0–A100, B0–B100, and C0–C100: The letters “A”, “B”, and “C” represent *L. bicolor* leaves, *P. viviparum* leaves, and *P. viviparum* bulbils, respectively. The numbers (0, 20, 40, 60, 80, and 100) following each letter (A, B, and C) indicate the percentage of tannin‐rich forages in the substrates.

Abbreviations: IVDMD, in vitro dry matter digestibility; MCP, microbial protein; NH_3_‐N, ammonia.

*p < 0.05, **p < 0.01, ***p < 0.001.

**FIGURE 5:**
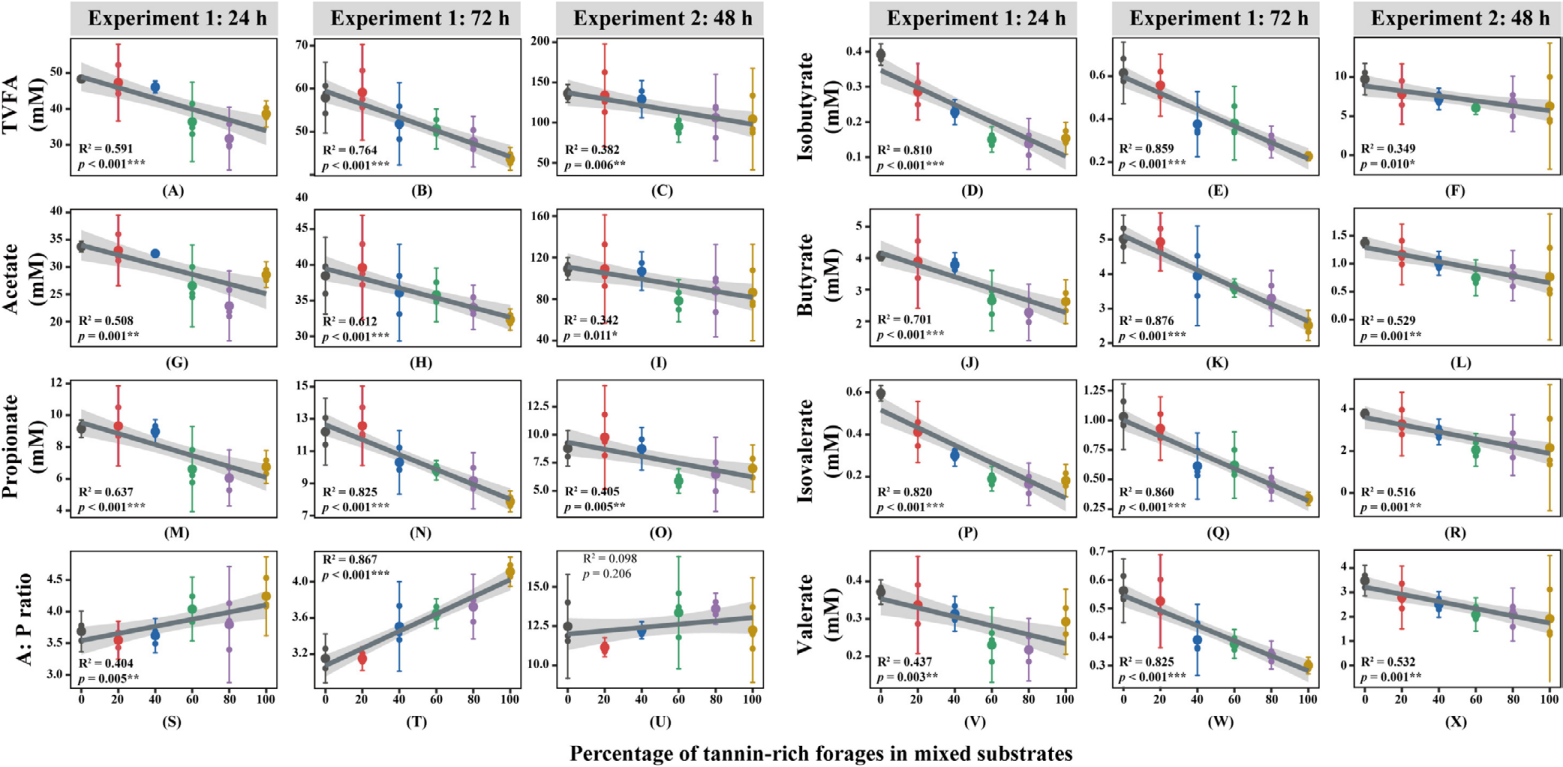
Linear correlations between the percentage of tannin‐rich forages in substrates and the concentrations of volatile fatty acids. The shaded part of the figure is the band of the 95% confidence interval. The smaller dots in panels represent the data of the replicates, and the larger dots represent the average of the data. The *p* and *R* ^2^ values in each panel were derived from linear regression. *p < 0.05, **p < 0.01, ***p < 0.001. A:P ratio, acetate to propionate ratio; TVFA, total volatile fatty acids.

As shown in Figure 5 and Table 6, the concentrations of TVFA and six individual VFA (acetate, propionate, butyrate, isobutyrate, valerate, and isovalerate) in the fermentation liquids were negatively correlated with the proportion of TRF in the substrates in Experiments 1 and 2 (*p* < 0.05). In Experiment 1, the acetate to propionate ratio (A:P ratio) was positively correlated with the proportion of *L. bicolor* leaves in the substrates (*p* < 0.01), whereas the ratio was not affected by the mixing ratios in Experiment 2 (*p* > 0.05).

**TABLE 6:** Effects of tannin‐rich forages mixed with alfalfa on volatile fatty acid concentrations in the fermentation.

		Treatments						*p*
Experiment 1		A0	A20	A40	A60	A80	A100	
24 h	TVFA (mM)	48.3 ± 0.08 a	47.3 ± 2.49 ab	46.1 ± 0.39 ab	36.4 ± 2.57 ab	31.7 ± 2.04 b	38.6 ± 0.85 ab	0.013*
	Acetate (mM)	33.7 ± 0.23 a	33.0 ± 1.51 a	32.5 ± 0.14 a	26.6 ± 1.74 b	22.9 ± 1.49 c	28.6 ± 0.55 b	< 0.001***
	Propionate (mM)	9.15 ± 0.128	9.33 ± 0.586	8.98 ± 0.175	6.61 ± 0.623	6.05 ± 0.411	6.75 ± 0.243	0.018*
	Isobutyrate (mM)	0.39 ± 0.007 a	0.29 ± 0.019 b	0.23 ± 0.008 c	0.15 ± 0.008 d	0.14 ± 0.017 d	0.15 ± 0.010 d	< 0.001***
	Butyrate (mM)	4.06 ± 0.033 a	3.89 ± 0.344 a	3.79 ± 0.089 a	2.66 ± 0.222 b	2.28 ± 0.207 b	2.62 ± 0.161 b	< 0.001***
	Isovalerate (mM)	0.60 ± 0.008 a	0.41 ± 0.034 b	0.30 ± 0.012 c	0.19 ± 0.014 d	0.16 ± 0.023 d	0.18 ± 0.018 d	< 0.001***
	Valerate (mM)	0.37 ± 0.008 a	0.34 ± 0.030 ab	0.31 ± 0.011 ab	0.23 ± 0.023 c	0.22 ± 0.019 c	0.29 ± 0.020 b	0.001**
	Acetate: Propionate	3.69 ± 0.075 bc	3.55 ± 0.069 c	3.62 ± 0.063 c	4.04 ± 0.118 ab	3.79 ± 0.214 bc	4.24 ± 0.145 a	0.015*
72 h	TVFA (mM)	57.9 ± 1.91 a	59.1 ± 2.59 a	51.8 ± 2.22 b	50.7 ± 1.08 b	47.7 ± 1.37 bc	43.7 ± 0.61 c	< 0.001***
	Acetate (mM)	38.5 ± 1.25 ab	39.6 ± 1.70 a	36.1 ± 1.57 abc	35.8 ± 0.88 bcd	34.1 ± 0.72 cd	32.3 ± 0.34 d	0.009**
	Propionate (mM)	12.21 ± 0.482 a	12.58 ± 0.572 a	10.31 ± 0.458 ab	9.81 ± 0.141 ab	9.17 ± 0.405 ab	7.87 ± 0.152 b	0.011*
	Isobutyrate (mM)	0.61 ± 0.033 a	0.56 ± 0.033 a	0.38 ± 0.035 b	0.39 ± 0.040 b	0.30 ± 0.017 bc	0.23 ± 0.005 c	< 0.001***
	Butyrate (mM)	5.02 ± 0.160 a	4.93 ± 0.195 a	4.00 ± 0.334 b	3.64 ± 0.061 bc	3.34 ± 0.186 c	2.56 ± 0.101 d	< 0.001***
	Isovalerate (mM)	1.03 ± 0.065 a	0.93 ± 0.062 a	0.63 ± 0.065 b	0.64 ± 0.065 b	0.47 ± 0.033 bc	0.35 ± 0.013 c	< 0.001***
	Valerate (mM)	0.56 ± 0.026 a	0.53 ± 0.038 a	0.40 ± 0.029 b	0.38 ± 0.012 b	0.34 ± 0.011 bc	0.31 ± 0.007 c	< 0.001***
	Acetate:Propionate	3.15 ± 0.062 d	3.15 ± 0.030 d	3.51 ± 0.115 c	3.65 ± 0.038 bc	3.72 ± 0.083 b	4.11 ± 0.038 a	< 0.001***

Experiment 2		B0	B20	B40	B60	B80	B100	
48 h	TVFA (mM)	136.2 ± 2.58 a	133.9 ± 14.85 a	129.2 ± 5.37 a	95.2 ± 4.55 b	106.2 ± 12.51 ab	104.4 ± 14.66 ab	0.062
	Acetate (mM)	109.1 ± 2.49 a	109.0 ± 12.13 a	106.9 ± 4.30 a	78.4 ± 4.75 b	88.0 ± 10.34 ab	86.3 ± 10.78 ab	0.076
	Propionate (mM)	8.78 ± 0.367 ab	9.77 ± 1.068 a	8.74 ± 0.443 ab	5.88 ± 0.253 c	6.47 ± 0.769 c	7.00 ± 0.485 bc	0.005**
	Isobutyrate (mM)	9.73 ± 0.466 a	7.82 ± 0.895 ab	7.19 ± 0.319 ab	6.05 ± 0.191 b	6.58 ± 0.822 b	6.28 ± 1.871 b	0.137
	Butyrate (mM)	1.37 ± 0.021 a	1.17 ± 0.126 ab	1.01 ± 0.050 abc	0.75 ± 0.074 c	0.79 ± 0.104 bc	0.76 ± 0.261 bc	0.027*
	Isovalerate (mM)	3.77 ± 0.048 a	3.29 ± 0.351 ab	2.91 ± 0.141 abc	2.05 ± 0.182 c	2.28 ± 0.335 bc	2.15 ± 0.697 c	0.028*
	Valerate (mM)	3.48 ± 0.146 a	2.79 ± 0.300 ab	2.50 ± 0.121 b	2.09 ± 0.159 b	2.09 ± 0.253 b	1.92 ± 0.600 b	0.032*
	Acetate:Propionate	12.49 ± 0.765 ab	11.16 ± 0.140 b	12.24 ± 0.127 ab	13.36 ± 0.828 a	13.61 ± 0.229 a	12.26 ± 0.773 ab	0.100

*Note:* Different lowercase letters indicate significant differences (*p* < 0.05) among growth stages. A0–A100, B0–B100, and C0–C100: The letters “A”, “B”, and “C” represent *L. bicolor* leaves, *P. viviparum* leaves, and *P. viviparum* bulbils, respectively. The numbers (0, 20, 40, 60, 80, and 100) following each letter (A, B, and C) indicate the percentage of tannin‐rich forages in the substrates.

Abbreviation: TVFA, total volatile fatty acid.

*p < 0.05, **p < 0.01, ***p < 0.001.

## 4 Discussion

### 4.1 Gas Production

Gas production of each treatment increased rapidly in the early fermentation stage and became stable in the later stage (Figure 1). This trend was consistent with those in other studies (Lagrange et al. 2019; Afzalani et al. 2022). The high gas production rate at the early stage may be due to the high content of fibers and CP in the substrates. During fermentation, the organic compounds were gradually depleted, resulting in a stable gas production rate in the later stage (Awati et al. 2006).

In Experiments 1 and 3, gas production declined linearly as the percentage of TRF in the substrates increased (Figure 2A,B,D), which agrees with other studies and proves our first hypothesis (Getachew et al. 2008; Tan et al. 2011). Tannins can bind fibers and proteins via hydrogen bonding to form complexes, which protect these nutrients from degradation by rumen microorganisms, as evidenced by the reduced IVDMD (Figure 4M,N; Patra and Saxena 2011; Rira et al. 2015). The reduced degradability of feeds contributed to less gas production. Tannins can also inhibit cellulolytic microorganisms and the activities of fibrolytic enzymes, which are primarily responsible for fermenting carbohydrates into VFAs and gases (Patra and Saxena 2011).

Different from Experiments 1 and 3, Experiment 2 showed no significant linear relationship between gas production and TRF proportion (Figure 2C). This discrepancy proves our second hypothesis and may be explained by differences in the chemical composition of the substrates. As the TRF percentage increased, the CP content in the substrates decreased slightly in Experiment 2, whereas it decreased sharply in Experiments 1 and 3 (Table 2). The rapid decrease in CP content led to a quick reduction in fermentable substrates, resulting in a decline in gas production (Gasmi‐Boubaker et al. 2005).

Overall, gas production is determined by a combination of factors, among which the dominant factor may be the tannin content and chemical composition of forages.

### 4.2 Methane Production

In Experiment 1, as the proportion of TRF in the substrates increased, CH_4_ production decreased linearly (Figure 3), which agrees with other studies and proves our first hypothesis (Puchala et al. 2005; Jayanegara et al. 2012; Rira et al. 2015). Tannins have been proved to inhibit CH_4_ production by reducing methanogenic populations and/or associated protozoal populations in the rumen (Bhatta et al. 2009). Tannins may also reduce CH_4_ emissions by retarding the degradation of fibers in the rumen, as supported by the reduced IVDMD, acetate, and butyrate production in our study (Figures 4M,N and 5G,H,J,K; Tavendale et al. 2005). In this study, only *L. bicolor* reduced total CH_4_ production (Table 4). In contrast, supplementation with *P. viviparum* leaves and its bulbils at varying levels did not significantly affect CH_4_ production (Table 4). The possible reason is that the anti‐methanogenic effect of CT is species‐specific, which is consistent with our second hypothesis (Aboagye and Beauchemin 2019).

In addition to tannins, fibers in forages may also exert influences on CH_4_ production. A meta‐analysis indicated that as fiber digestibility or the amount of digested fiber increased, the CH_4_ yield per unit of digested fiber decreased (Clauss et al. 2020). Compared to *P. viviparum* , *L. bicolor* contained more NDF, leading to less CH_4_ yield.

### 4.3 In Vitro Fermentation Parameters

The MCP content in Experiment 2 was reduced linearly as the proportion of *P. viviparum* leaves in the substrates increased (Figure 4G), probably because CT can inhibit microbial growth and enzyme activities in the rumen (Castro‐Montoya et al. 2018). In Experiments 1 and 2, NH_3_‐N content decreased linearly with increasing tannin content in the substrates (Figure 4I–K). This decrease could be explained by tannins binding to rumen proteins and retarding protein degradation, or by tannins inhibiting the activity of microbial deaminase (Bhatta et al. 2009; Ghelichkhan et al. 2018). Ammonia, isobutyrate, and isovalerate are the breakdown products of amino acids during fermentation, and the decline of the NH_3_‐N content in this study was supported by the decline of the isobutyrate and isovalerate concentrations (Figure 5D–F,P–R; Chen et al. 2021). Conversely, in Experiment 3, no linear relationship was observed between NH_3_‐N and CT content in the substrates (Figure 4L). Since the CT content of *P. viviparum* bulbils in Experiment 3 was lower than that in Experiments 1 and 2, a less pronounced effect on reducing NH_3_‐N content was observed.

In Experiment 1, as the proportion of TRF in the substrates increased, IVDMD decreased linearly owing to the increased binding of tannins to carbohydrates (Figure 4M,N; Jayanegara et al. 2019). However, in Experiments 2 and 3, no linear relationship was observed between IVDMD and CT content in the substrates (Figure 4O,P). This discrepancy could be attributed to the different rumen fluids used in these experiments. Reduced IVDMD led to less VFA production, which may increase pH in the fermentation liquids. On the other hand, the simultaneous reduction in NH_3_‐N appeared to decrease pH. As a result, the overall effects showed a pH decline. In this study, the changes in each fermentation parameter (pH, MCP, NH_3_‐N, or IVDMD) were not consistent among the three experiments, which proves our second hypothesis.

### 4.4 Volatile Fatty Acids

Volatile fatty acids are the major metabolites of carbohydrates in the rumen, which can be absorbed into blood circulation through the rumen wall and transported to tissues as energy sources to complete important biosynthetic functions. The VFA concentrations in the fermentation liquids were negatively correlated with the proportion of TRF in the substrates (Figure 5), which agrees with other studies (Paengkoum et al. 2015; Bueno et al. 2020). The decrease in VFA concentrations may be related to the formation of recalcitrant tannin‐carbohydrate and tannin‐protein complexes, or to the toxicity of tannins to rumen microorganisms, or to both (Hassanat and Benchaar 2013). Particularly, the decrease in the concentrations of valerate and isovalerate was attributed to the decline of CP content in the substrates. A strong positive correlation between the CP content and the concentrations of valerate and isovalerate was reported in another study, which showed that lower CP content led to less fermentable substrates and products (Getachew et al. 2004).

The change of the A:P ratio in Experiment 1 differed from that in Experiment 2. The A:P ratio was positively correlated with the proportion of TRF in the substrates in Experiment 1, whereas that was not affected by the TRF proportion in Experiment 2 (Figure 5S–U). The inconsistent results were also noticed in other studies. When tannins were added to diets, increased, decreased, or unchanged A:P ratios were all reported (Beauchemin et al. 2007; Tan et al. 2011; Aguerre et al. 2016; Aboagye et al. 2018; Zhang et al. 2025). In addition, supplementing quebracho CT extract in different forage diets led to contradictory results (Dschaak et al. 2011). The above results could be attributed to different content, molecular weights, and chemical structures of CT as well as different diets in those studies (Aboagye and Beauchemin 2019).

### 4.5 Donor Species and Methodological Caveats

The results obtained from different animal species are not completely consistent, which could be attributed to different rumen microbial populations originating from the evolutionary specialization of species (Bueno et al. 2015). In the present study, different donor animals were used. Specifically, Experiment 1 was based on yellow cattle, whereas Experiments 2 and 3 were based on small‐tailed Han sheep. Differences in the rumen environment among animal species may affect microbial populations and/or the activities of degrading enzymes, which lead to different fermentation characteristics. For example, substrate degradation and fermentation rates differed in rumen fluid between buffalo and sheep, and between buffalo and cattle (Calabrò et al. 2005, 2008). Moreover, rumen microorganisms from different species may exhibit varying tolerances or susceptibilities to plant secondary metabolites such as tannins (Waghorn 2008; Min and Solaiman 2018). Thus, the observed responses to TRF may partly reflect the distinct microbial characteristics of the donor animals. Therefore, caution must be taken when we compare the results among these experiments.

Apart from the above consideration, it is important to mention that in vitro and in vivo systems differ fundamentally, especially in terms of fermentation dynamics. The rumen functions as a continuous and adaptive environment, whereas the in vitro systems used in this study were static and did not fully replicate the complexity of microbial interactions in live animals. Therefore, when interpreting and generalizing the in vitro findings to in vivo conditions, caution should be taken.

### 4.6 Threshold for Mixing Ratio of Tannin‐Rich Forages and Alfalfa

We found that when the mixing ratio of TRF and alfalfa was below 40:60, the overall fermentation of the mixed substrates was improved compared to that of alfalfa alone. When the mixing ratio was above 40:60, the effects of mixed fermentation were unsatisfactory. Therefore, there might exist a threshold for the mixing ratio of TRF and alfalfa. Specifically, in Experiment 1, compared with alfalfa alone, *L. bicolor* mixed with alfalfa at a ratio below 40:60 showed lower gas production, CH_4_ yield, and NH_3_‐N content without affecting MCP content or IVDMD. Similarly, in Experiment 2, compared with alfalfa alone, *P. viviparum* leaves mixed with alfalfa at a ratio below 40:60 showed lower NH_3_‐N content without affecting IVDMD or VFA concentrations. In Experiment 3, compared with alfalfa alone, *P. viviparum* bulbils mixed with alfalfa at a ratio below 40:60 showed lower NH_3_‐N content without affecting MCP content or IVDMD. The optimal mixing ratios of TRF and alfalfa reported in several studies are also within this threshold. For instance, mixing *Onobrychis viciifolia* and/or *Lotus corniculatus* with alfalfa in a diet at a 30:70 ratio reduced bloat and NH_3_‐N formation in sheep (Lagrange et al. 2019). However, the optimal mixing ratios in some studies exceed this threshold. For example, when *O. viciifolia* was mixed with alfalfa at a 75:25 ratio and fed to sheep, nitrogen retention was the greatest in small intestines and nitrogen rumen degradability was the lowest (Aufrère et al. 2013). This discrepancy may be due to different tannin sources and animal species, leading to varying effects on the digestive processes of ruminants (Aboagye et al. 2018).

Condensed tannins are generally considered beneficial to animals when the CT content is less than 50 g kg^−1^ DM. High content of CT can reduce DM intake and produce multiple anti‐nutritional and toxic effects (Mueller‐Harvey 2006). In the present study, the adverse effects on rumen fermentation were observed when the CT content in the substrates exceeded 100.1, 72.6, and 25.5 g kg^−1^ DM for *L. bicolor* leaves, *P. viviparum* leaves, and *P. viviparum* bulbils, respectively (Table 2). This finding highlights that the critical tannin level is not universal among forage materials, depending on tannin sources, content, and biological activities. Therefore, caution should be made when generalizing the “safe limits” of tannins across different forage sources.

## 5 Conclusions

In this study, we investigated the effects of *L. bicolor* and *P. viviparum* mixed with alfalfa at different ratios on in vitro rumen fermentation. *L. bicolor* showed a more prominent effect on fermentation than *P. viviparum* when mixed with alfalfa. This indicates that different tannin‐rich forages have various effects on in vitro fermentation. In addition, when the mixing ratio of tannin‐rich forages and alfalfa was below the threshold of 40:60, the overall fermentation of the mixed substrates was improved compared to that of alfalfa alone. The results of this study will support the application of *L. bicolor* and *P. viviparum* in ruminant diets. In practical livestock production, incorporating tannin‐rich forages into alfalfa‐based diets at appropriate ratios may improve ruminal fermentation, increase nutrient utilization, and mitigate methane emissions and environmental pollution. This provides a specific forage use strategy with both economic benefits and environmental advantages in arid and semiarid grasslands and alpine meadows.

## Author Contributions

**Jieqi Zhang:** conceptualization (equal), data curation (equal), formal analysis (lead), software (equal), visualization (lead), writing – original draft (lead), writing – review and editing (lead). **Yongsheng He:** formal analysis (equal), investigation (equal), methodology (equal), resources (equal). **Zifan Chen:** formal analysis (equal), investigation (equal), methodology (equal), resources (equal). **Fujiang Hou:** funding acquisition (equal), writing – review and editing (equal). **Ding Guo:** conceptualization (equal), writing – review and editing (equal). **Jing Wang:** conceptualization (equal), funding acquisition (equal), project administration (lead), supervision (lead), writing – review and editing (lead).

## Conflicts of Interest

The authors declare no conflicts of interest.

## Supporting information


**Table S1:** Ingredients and chemical composition of the diets offered to the donor animals.
**Method S1:** Measurement of methane and volatile fatty acid concentrations.

## Data Availability

Data available on request from the authors.
