# Supplementary material for: Effects of Tannin‐Rich Forages ( Lespedeza bicolor and Polygonum viviparum ) Mixed With Medicago sativa on In Vitro Rumen Fermentation
Source: Food Sci Nutr. 2025 Oct 14;13(10):e71015. doi: 10.1002/fsn3.71015 (PMC12519511; doi:10.1002/fsn3.71015)
Supplement: Supplementary file 1 — Table S1: Ingredients and chemical composition of the diets offered to the donor animals. Method S1: Measurement of methane and volatile fatty acid concentrations. [file FSN3-13-e71015-s001.docx]

**Supporting Information**

**Article title:** Effect of tannin-rich forages (*Lespedeza bicolor* and *Polygonum viviparum*) mixed with *Medicago sativa* on the *in vitro* rumen fermentation.

**Authors:** Jieqi Zhang, Yongsheng He, Zifan Chen, Fujiang Hou, Ding Guo*, Jing Wang*

**Table S1** Ingredients and chemical composition of the diets offered to the donor animals.

| Experiment 1 | | Experiment 2 and 3 | |
| --- | --- | --- | --- |
| Donor animal: yellow cattle | | Donor animal: small-tailed Han sheep | |
| Ingredients (g kg^-1^ DM) | | Ingredients (g kg^-1^ DM) | |
| Maize silage | 500 | Oat hay | 528 |
| Corn grain | 214.6 | Corn | 201 |
| Sprayed corn skin | 180.5 | Corn starch | 112 |
| Soybean meal | 67.4 | Sucrose | 29.8 |
| Wheat bran | 17.5 | Soybean meal | 26.8 |
| Sodium bicarbonate | 12 | Wheat bran | 24.2 |
| Sodium chloride | 8 | Cotton seed meal | 22.7 |
|  |  | Molasses | 22.1 |
|  |  | Calcium hydrophosphate | 12.1 |
|  |  | Sodium chloride | 10.7 |
|  |  | Soybean oil | 6.5 |
|  |  | Calcium carbonate | 4.1 |
| Chemical composition (g kg^-1^ DM) | | Chemical composition (g kg^-1^ DM) | |
| Crude protein | 104.8 | Crude protein | 128.9 |
| Neutral detergent fibre | 477.1 | Neutral detergent fibre | 347.6 |
| Acid detergent fibre, | 211.2 | Acid detergent fibre | 176.3 |

**Method S1** Measurement of methane and volatile fatty acid concentrations

Methane produced during fermentation was analyzed using a gas chromatography instrument (GC, HP4890D, Agilent Co., Santa Clara, CA, USA) equipped with a hydrogen flame ionization detector. Chromatographic separation was conducted using a stainless steel column packed with 13XMS (2 m × 3 mm I.D., 60-80 mesh, Buchem B.V., Apeldoorn, Netherlands). The temperatures of the injector and the column oven were set at 200°C and 55°C, respectively. Ultra-pure nitrogen (99.999%) was used as the carrier gas at a flow rate of 30 ml min^-1^. Calibration was performed using a certified methane standard (4.81 µl L^-1^) provided by the National Institute of Metrology, China.

The determination of volatile fatty acid concentrations was carried out using a GC instrument (ThermoQuest 8000top, Italia SpA, Rodano, Milan, Italy) (Getachew et al., 2001). Chromatographically pure acetate, propionate, butyrate, isobutyrate, valerate, and isovalerate were used as standards, with chromatographically pure crotonic acid serving as an internal standard. The analysis was conducted using a fused silica capillary column (FFAP; 30 m × 0.25 mm I.D. × 0.25 µm, Rodano, Milan, Italy).

**References**

Getachew, G., DePeters, E. J., Robinson, P. H., & Taylor, S. J. (2001). *In vitro* rumen fermentation and gas production: influence of yellow grease, tallow, corn oil and their potassium soaps. *Animal Feed Science and Technology*, *93*(1-2), 1-15. https://doi.org/10.1016/S0377-8401(01)00264-4
